# Improving saliva shotgun metagenomics by chemical host DNA depletion

**DOI:** 10.1186/s40168-018-0426-3

**Published:** 2018-02-27

**Authors:** Clarisse A. Marotz, Jon G. Sanders, Cristal Zuniga, Livia S. Zaramela, Rob Knight, Karsten Zengler

**Affiliations:** 1 Department of Pediatrics, University of California San Diego, La Jolla, CA USA; 2 Center for Microbiome Innovation, University of California San Diego, La Jolla, CA USA; 3 Department of Computer Science and Engineering, University of California San Diego, La Jolla, CA USA

**Keywords:** Microbiome, Host depletion, Microbial enrichment, Propidium monoazide, Shotgun sequencing, Saliva

## Abstract

**Background:**

Shotgun sequencing of microbial communities provides in-depth knowledge of the microbiome by cataloging bacterial, fungal, and viral gene content within a sample, providing an advantage over amplicon sequencing approaches that assess taxonomy but not function and are taxonomically limited. However, mammalian DNA can dominate host-derived samples, obscuring changes in microbial populations because few DNA sequence reads are from the microbial component. We developed and optimized a novel method for enriching microbial DNA from human oral samples and compared its efficiency and potential taxonomic bias with commercially available kits.

**Results:**

Three commercially available host depletion kits were directly compared with size filtration and a novel method involving osmotic lysis and treatment with propidium monoazide (lyPMA) in human saliva samples. We evaluated the percentage of shotgun metagenomic sequencing reads aligning to the human genome, and taxonomic biases of those not aligning, compared to untreated samples. lyPMA was the most efficient method of removing host-derived sequencing reads compared to untreated sample (8.53 ± 0.10% versus 89.29 ± 0.03%). Furthermore, lyPMA-treated samples exhibit the lowest taxonomic bias compared to untreated samples.

**Conclusion:**

Osmotic lysis followed by PMA treatment is a cost-effective, rapid, and robust method for enriching microbial sequence data in shotgun metagenomics from fresh and frozen saliva samples and may be extensible to other host-derived sample types.

**Electronic supplementary material:**

The online version of this article (10.1186/s40168-018-0426-3) contains supplementary material, which is available to authorized users.

## Background

In the past decade, sequencing costs have plummeted, and 16S rRNA gene amplicon sequencing has become a nearly ubiquitous tool used to characterize bacterial populations from a wide range of environments and host systems [1, 2]. This technique has revealed that bacteria inhabit a far greater range of human body sites than previously believed, including many long presumed to be sterile (e.g., urine [3], breast milk [4], blood [5], and atherosclerotic plaque [6]). However, 16S rRNA gene amplicon sequencing has several limitations.

Taxonomic resolution is intrinsically limited in amplicon analysis and can fail to distinguish species and strains with distinct biological functions. Primer choice can affect the representation of particular clades of bacteria [7]. Eukaryotic microbes are not captured by 16S rRNA gene amplicon sequencing and require 18S rRNA gene, internal transcribed spacer, or mitochondrial sequencing approaches; viruses are not detected by any of these methods and require custom clade-specific primers.

**Fig. 1:**
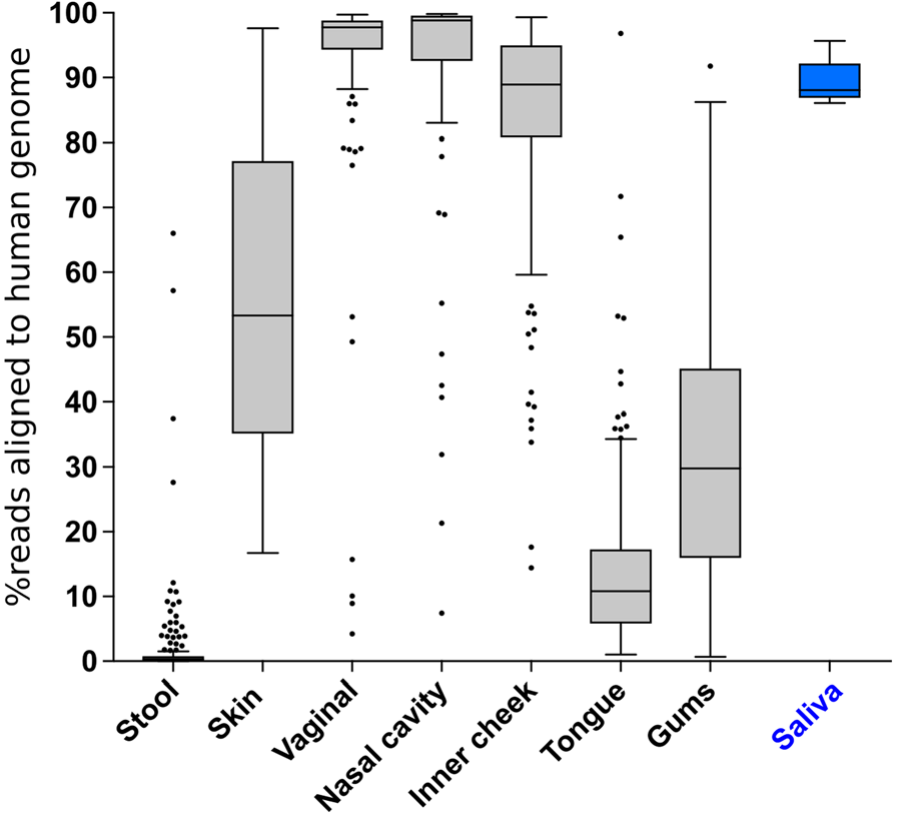
Percent of shotgun metagenome sequencing reads aligning to human genome varies by sample type. Data from the Human Microbiome Project (HMP; black) of healthy individuals demonstrates the percentage of human reads by sample type. Saliva data (blue) was collected from healthy individuals in this study. Stool *n* = 249, skin *n* = 29, vaginal *n* = 103, nasal cavity *n* = 112, inner cheek *n* = 175, tongue *n* = 208, gums *n* = 189, and saliva *n* = 24 (this study)

Shotgun metagenomic sequencing overcomes these hurdles because it analyzes total DNA extracted from a sample and does not depend on target-specific primers. For the analysis of host-derived samples, this advantage of shotgun sequencing is also its vulnerability. Because the human genome is roughly one thousand times larger than an average bacterial genome (~ 3 × 10^9^ versus ~ 3 × 10^6^ bp), host DNA can quickly drown out microbial reads in samples containing even a relatively small number of human cells. The proportion of human cells to microbial cells varies widely by sampling site, and consequently, the percentage of shotgun sequencing reads aligning to the human genome varies widely by sampling site. For example, fecal samples from healthy controls typically yield < 10% human genome-aligned reads, but human saliva, nasal cavity, skin, and vaginal samples routinely contain > 90% (Fig. 1). Therefore, identifying a method to reproducibly deplete host reads for shotgun sequencing is crucial for almost all host-derived microbiome studies.

Current approaches to deplete host reads can be divided into two major groups: those that act prior to DNA extraction (pre-extraction) and those that act on DNA after extraction (post-extraction). Pre-extraction approaches generally follow a two-step procedure. The first step is to selectively lyse mammalian cells, which is easy because the mammalian cell membrane is more fragile than most microbial membranes/cell walls. The second step removes exposed DNA enzymatically, leaving only the intact microbial cells for downstream analysis. These kits have improved microbial sequencing yield in a variety of sample types, including bronchoalveolar lavage fluid [8], blood [9], and sonicate fluid from prosthetic joint infections [10]. However, the multiple wash steps required limit the potential of low biomass samples to be successfully treated [11]. Furthermore, loss of bacterial DNA and a potential bias toward Gram-positive taxa has been reported [12].

Post-extraction separation approaches avoid some of these hurdles and thus pose an attractive alternative. One approach targets methylated nucleotides [13], which are typically more frequent in eukaryotic genomes. However, a bias against microbes with AT-rich genomes has been reported [14], and therefore, this method is not suitable for eukaryotic microorganisms with methylation patterns similar to the host. Another approach is targeting host-specific sequences for hybridization-based depletion with CRISPR/Cas9. This method has been successfully employed for highly repetitive rRNA sequences [15] but is not easily adapted to depletion of sequences at the genome scale.

To overcome the disadvantages associated with each of the currently available host-depletion methods, we optimized a cost-effective technique with minimal sample processing and hands-on time. Similar to other pre-extraction methods, it starts with selective mammalian cell lysis, but instead of enzymatic digestion of exposed DNA, we employed propidium monoazide (PMA). PMA has been used extensively over the past decade for detection of live/dead cells [16]. Similar to propidium iodide, PMA is a cell membrane impermeable DNA intercalator. Upon exposure to visible light, the azide group of the PMA molecule is photolytically cleaved and undergoes a C–H insertion reaction to form a covalent bond with DNA [17]. It is thought that this reaction fragments the DNA, effectively eliminating any exposed DNA from downstream analysis [17, 18]. Any excess PMA in the sample reacts with water and becomes inert. We induced selective osmotic lysis of mammalian cells by resuspension in pure water followed by treatment with PMA (lyPMA). This method requires less than 5-min hands-on time and involves no special laboratory equipment.

To evaluate the efficiency and potential bias of the lyPMA treatment, we compared this protocol to raw samples and four alternative methods used for host depletion: 5-μm filtration (Fil), QIAamp DNA Microbiome Kit (QIA), MolYsis™ Basic (Mol), and NEBNext® Microbiome DNA Enrichment Kit (NEB). We chose saliva samples to compare methods of host depletion because it is easy to collect, has enough biomass to be divided into multiple groups per sample, and consistently has a high (~ 90%) percentage of human DNA in shotgun metagenomic sequencing (Fig. 1). The efficiency of host depletion and the effect on microbial community was assessed.

## Results

### Differential cell size-based approaches to host DNA depletion

One of the most obvious differences between mammalian and microbial cells is their size. Our preliminary attempts to reduce host DNA therefore focused on separating cells according to size. Because buccal epithelial cells are on average 50 μm wide, whereas a typical bacterial coccus is ~ 1 μm, we passed saliva samples across a 5-μm filter and analyzed the filtrate and residue compared to the raw sample. We designed a qPCR assay to evaluate the percentage of host DNA relative to untreated sample using a human-specific primer against the PTGER2 gene [19]. No significant difference across any of these three partitions was observed (Additional file 1: Figure S1A). To exclude the potential of host cell shearing during filtration, we next tried differential centrifugation to enrich for microbial DNA. First, a short, slow centrifugation (30 s at 2500*g*) of human saliva was performed to pellet large cells [20], and then the supernatant was washed with a longer, faster centrifugation (8 min at 10,000*g*) to pellet all remaining cells. No significant difference in percentage of human DNA at any of these steps compared to the original raw sample was observed (Additional file 1: Figure S1B). Lastly, we attempted to take advantage of differences in forward and backward scatter (which canonically correlates to event size and density, respectively) using flow cytometry to separate microbial from human cells. Although three distinct groups of varying size were clearly observed (Additional file 1: Figure S1C), there was no significant difference in percentage of human DNA among the sorting gates compared to the raw sample (Additional file 1: Figure S1D).

These preliminary attempts to separate mammalian from microbial cells based on cell size were unsuccessful in reducing the amount of host DNA. We hypothesized that there must be a significant amount of extracellular host DNA that is not separated by size-based approaches. Indeed, DNAse treatment of saliva samples after a short, slow centrifugation significantly reduced the percentage of human DNA (Additional file 1: Figure S1E). However, enzymatic treatment can be expensive, sensitive, and, because it must be processed on fresh samples, difficult to scale. As an alternative to enzymatic digestion of extracellular DNA, we next tested the ability of PMA to remove host DNA.

### Optimization of lyPMA for host DNA removal

To optimize the lyPMA protocol, we compared different methods of selective mammalian cell lysis and multiple concentrations of PMA. Quantitative polymerase chain reaction (qPCR) analysis of the human-specific PTGER2 gene revealed that, compared to untreated controls, PMA treatment reduced the percentage of human DNA following selective mammalian cell lysis by sonication (25.6%) and osmotic lysis with H2O (1.7%) and following mammalian cell removal by differential centrifugation (1.4%) (Additional file 2: Figure S2A). Interestingly, PMA treatment of raw saliva sample (without an initial selective lysis step) also reduced the percentage of human DNA (16.8%), suggesting that the majority of human DNA in saliva is already exposed. We also evaluated the effect of PMA concentration (1, 10, and 50 μM) on the reduction of extracellular host DNA following differential centrifugation. Treatment with 10 μM PMA was the optimal concentration to achieve host DNA reduction without compromising microbial DNA recovery, although the results were not highly sensitive to PMA concentration (Additional file 2: Figure S2B). The relative percentage of bacterial DNA was also evaluated by qPCR to ensure specific removal of human DNA. Compared to raw saliva samples, osmotic lysis (7.82-fold) and 10 μm PMA concentration (13.4-fold) had the greatest increase in the proportion of bacterial DNA (Additional file 2: Figure S2C, D). We thus used osmotic lysis followed by 10 μM PMA treatment for comparison of the lyPMA approach to commercially available alternatives.

### Efficiency of host depletion across microbial enrichment methods

To compare lyPMA to other methods of host DNA depletion, saliva samples were collected from eight healthy participants (4 mL each). Each sample was homogenized and divided into 18 separate 200-μl aliquots. Triplicate aliquots were processed in parallel for each of the six methods (i.e., untreated (raw) samples, Fil, NEB, Mol, QIA, and lyPMA; see the “Methods” section). DNA was extracted from all samples, and shotgun DNA sequencing libraries were prepared in parallel. The concentration of DNA following host depletion was significantly lower in all five methods compared to raw samples (Additional file 3: Figure S3A). Samples for sequencing were pooled such that the raw samples had twice as many reads compared to the host-depleted samples, with the assumption that we could thereby increase the number of microbial reads in the raw samples to better assess potential taxonomic biases (Additional file 3: Figure S3B). Samples with less than 50,000 quality-filtered microbial reads (*n* = 7) were excluded from downstream analysis, leaving 137 samples to evaluate efficiency of host depletion and microbial community effect.

**Fig. 2:**
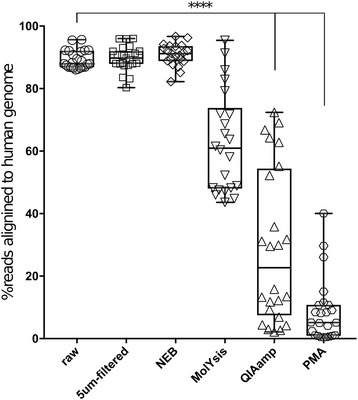
Host DNA depletion in saliva reduces the percentage of sequencing reads aligning to the human genome. Saliva was collected from eight individuals and divided into triplicate aliquots for each of the processing methods. The fraction of quality filtered shotgun sequencing reads mapping to the human genome was assessed with Bowtie 2. One-way ANOVA with Tukey’s multiple comparison correction, significance *p* < 0.0001

The percentage of shotgun metagenomic sequencing reads mapping to the human genome in each sample was evaluated using Bowtie 2. The average percentage of human reads in the raw samples (89.29 ± 0.61%) was no different than that in samples filtered across a 5-μm filter (89.69 ± 0.84%) or samples treated with the NEB kit (90.83 ± 0.77%) (Fig. 2). Treatment with Mol (62.88 ± 3.46%), QIA (29.17 ± 5.04), and lyPMA (8.53 ± 2.08%) all significantly depleted host reads compared to the raw samples (one-way ANOVA with Tukey’s multiple comparison *p* < 0.0001). These three methods were all significantly different from each other, with lyPMA performing the best followed by QIA and then Mol (*p* < 0.0001).

### Effect on microbial community composition caused by host depletion methods

**Fig. 3:**
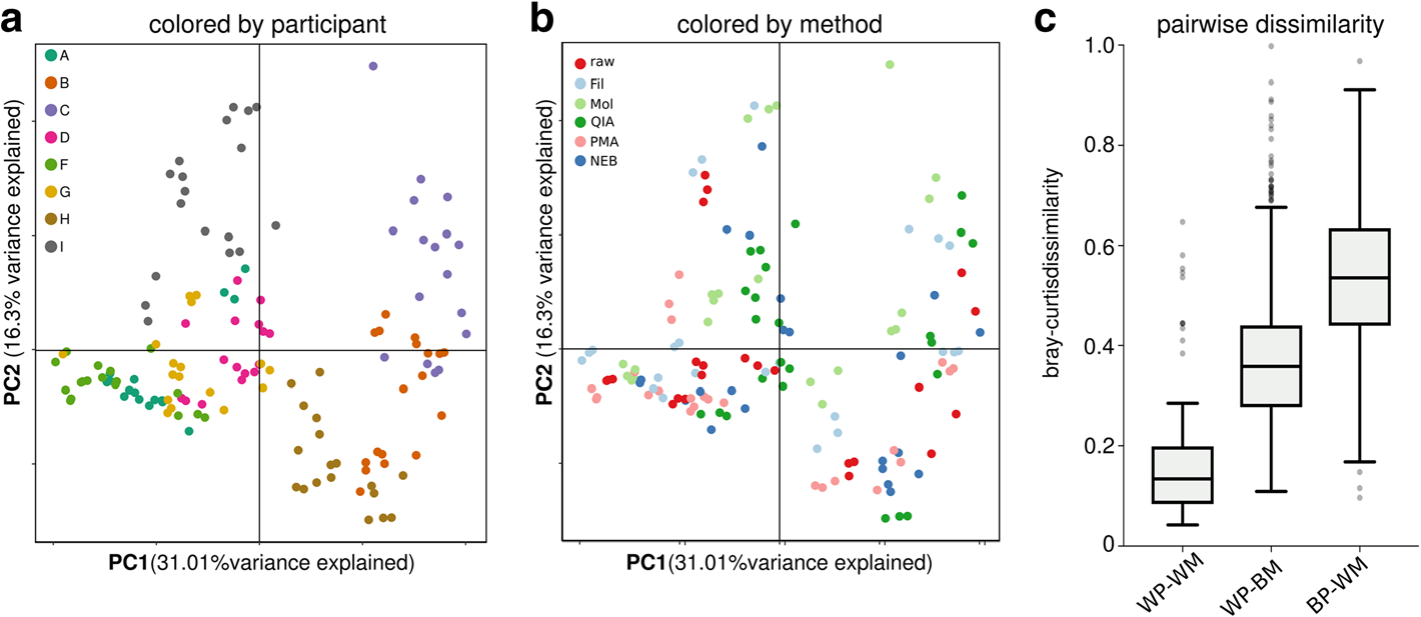
Differences in saliva microbiome driven by participant and not method of host depletion. Microbial reads cluster by participant (**a**) and not method of host depletion (**b**) in PCoA space using Bray-Curtis distance. **c** Pairwise Bray-Curtis dissimilarities: within participant, within method (WP-WM); within participant, between methods (WP-BM); and between participants, within methods (BP-WM). Each category is statistically significantly different from each other group (Kruskal-Wallis with Benjamini and Yekutieli FDR correction *p* < 0.0001)

Each participant had a distinct pattern of microbial genera that altered slightly upon host depletion (Additional file 4: Figure S4). In principal coordinates analyses (PCoA) [21], samples cluster by participant and not method of host depletion (Fig. 3a, b), suggesting that differences in the microbial community were driven more by biological differences among participants rather than technical effects from any of the host depletion methods. Indeed, Bray-Curtis (BC) dissimilarities between samples from different participants processed with the same host depletion method were significantly greater than dissimilarities from the same participant processed with different host depletion methods (Fig. 3c). This held true across other beta-diversity metrics including phylogeny-informed weighted and unweighted UniFrac (Table 1). Furthermore, relative abundances of microbial taxa within participants among host depletion methods were tightly correlated with few obvious outliers and Spearman’s rank correlation coefficient ≥ 0.75 (Additional file 5: Figure S5). To evaluate whether differences in read depth affected these results, we subsampled 50,000 non-human reads from each sample and found similar results, namely that the participant explained more of the variability in microbial taxa than method of host DNA depletion (Adonis of Bray-Curtis distance *R*^2^ = 0.169 by method, 0.556 by participant, F.model= 15.152 by method, 36.614 by participant; *p* value < 0.001 for all).

**Table 1:** Adonis statistical assessment of beta-diversity metrics driven by participant or host DNA depletion method

Beta-diversity metric	Variable	Degrees of freedom	*R* ^2^	F.model	*p* value
Unweighted UniFrac	Method	5	0.092	6.345	0.001
	Participant	7	0.548	26.932	0.001
Weighted UniFrac	Method	5	0.118	12.331	0.001
	Participant	7	0.644	47.947	0.001
Bray-Curtis	Method	5	0.149	14.444	0.001
	Participant	7	0.594	41.019	0.001
Binary Jaccard	Method	5	0.168	9.537	0.001
	Participant	7	0.396	16.059	0.001

**Fig. 4:**
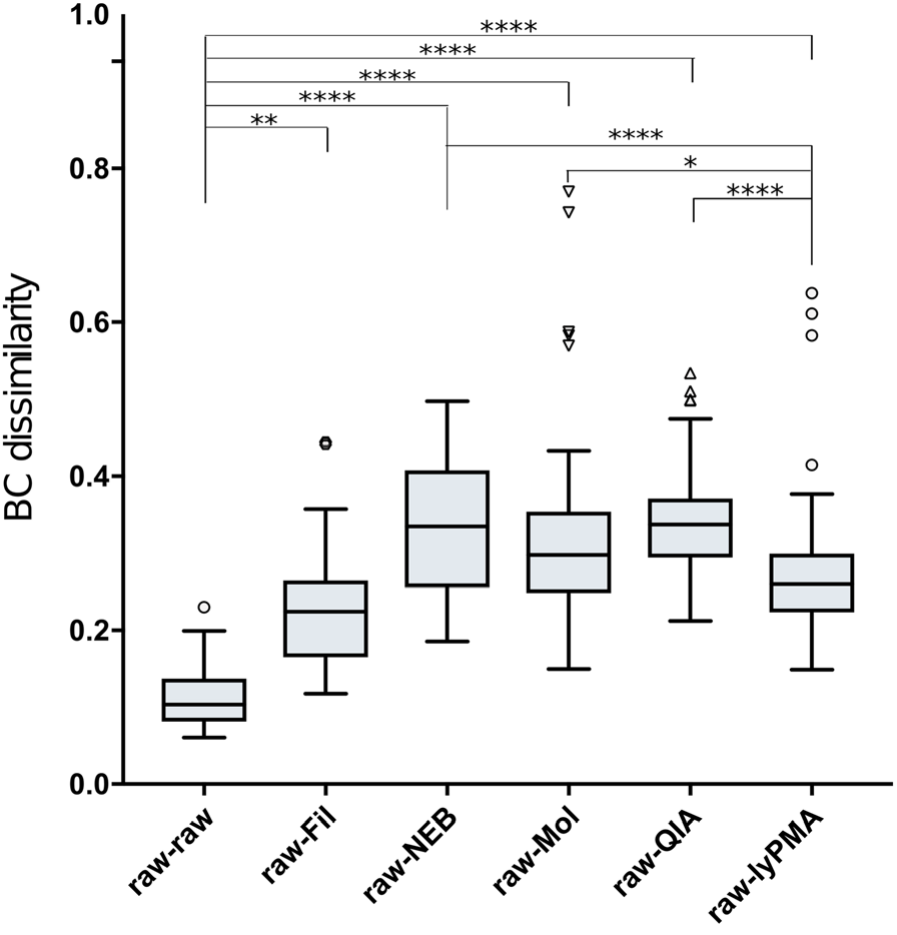
Bray-Curtis dissimilarity between host depleted and raw sample from the same participant. The pairwise Bray-Curtis dissimilarity value was calculated between each sample with every other sample in this study. The dissimilarity values between each sample and the matched participant raw sample are presented here. Statistical significance calculated with Kruskal-Wallis with Benjamini and Yekutieli FDR correction *p* < 0.05. raw-raw *n* = 22, raw-Fil *n* = 66, raw-NEB *n* = 63, raw-Mol *n* = 63, raw-QIA *n* = 69, raw-lyPMA *n* = 69

However, BC dissimilarities among host-depleted samples from the same participant were significantly higher than noise from technical replication (within raw triplicate samples), indicating that there is a significant effect of host depletion on microbial community composition (Fig. 3c). We then compared the BC dissimilarities between each method of host depletion and the corresponding raw samples (Fig. 4). Each of the five treatments had significantly greater BC dissimilarity than technical variation among raw replicates (0.115 ± 0.009; Kruskal-Wallis with Benjamini and Yekutieli FDR correction *p* < 0.05). However, lyPMA (0.273 ± 0.011) and Fil (0.226 ± 0.009) were significantly more similar to raw samples than NEB (0.333 ± 0.010), Mol (0.321 ± 0.015), and QIA (0.342 ± 0.008) (Kruskal-Wallis with Benjamini and Yekutieli FDR correction *p* < 0.05). There was no statistical difference observed among NEB, Mol, and QIA distance from corresponding raw samples. To look for bacteria affected by host DNA depletion, we performed a pairwise comparison of the relative abundance of each taxon in raw versus host DNA depletion method for each individual using *t* tests with Benjamini, Krieger, and Yekutieli false discovery rate correction at 1%. No taxa were identified to be consistently differentially abundant across the host DNA depletion methods.

### Evaluation of lyPMA treatment on frozen saliva samples

Microbiome sampling often requires samples to be frozen and preserved for downstream processing. We therefore tested the effectiveness of lyPMA on previously frozen samples. Saliva samples from three participants were divided into 200-μl aliquots and either immediately stored at − 20 °C or cryopreserved by mixing with 20% glycerol prior to freezing (Additional file 6: Figure S6). After 3 days, samples were thawed and replicate frozen and cryopreserved samples were treated with lyPMA. Similar to the freshly processed samples, the majority of reads from the untreated samples aligned to the human genome (84.73 ± 2.56%). lyPMA samples that were cryopreserved with glycerol had a similar reduction in host-aligned reads to the freshly processed samples (7.18 ± 3.09%). Without cryopreservation, lyPMA was less efficient and more variable (53.78 ± 27.43%). The BC dissimilarity value was similar for technical replicates of raw samples (0.146 ± 0.004) and raw versus matched cryopreserved lyPMA (0.276 ± 0.071) but was higher for raw versus matched non-cryopreserved lyPMA (0.348 ± 0.002).

## Discussion

**Fig. 5:**
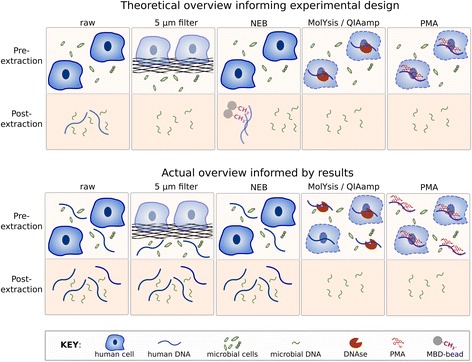
Experimental overview. A graphical summary of the experimental design and results

We compared the efficiency of five methods of host DNA depletion on human saliva for shotgun metagenomic sequencing as outlined in Fig. 5. Although filtering saliva across a 5-μm filter excludes intact host cells, no difference was observed in the percentage of host-aligned reads in DNA extracted from the filtrate. This is likely due to the high amount of extracellular DNA in saliva and explains why preliminary experiments based on separating microbial from host cells based off size (i.e., 5-μm filtration and flow cytometry) were unsuccessful.

In our hands, immunoprecipitation of methylated eukaryotic DNA was unsuccessful at reducing the percentage of host-aligned sequences, as evidenced by our post-extraction processing of DNA with the NEBNext enrichment kit. It is important to note that this protocol recommends an input of high molecular weight gDNA (> 15 kb fragments) and our input gDNA peaked at ~ 10 kb. To achieve maximal efficiency, samples should be extracted with phenol chloroform followed by isolation of appropriately sized DNA from a low-melt agarose gel [13]. However, we extracted all samples in parallel using a high-throughput DNA extraction pipeline [22] to reduce any confounding variables from differing DNA extraction methods.

Selective lysis of mammalian cells followed by removal of exposed DNA was a consistently effective method of reducing host-aligned sequencing reads. MolYsis, QIAamp, and lyPMA treatments all significantly improved microbial yield, with lyPMA outperforming alternative treatments. It is possible that increasing the enzyme concentration of the MolYsis or QIAamp kits would further reduce the percentage of host-aligned reads. Regardless, lyPMA treatment has an advantage over enzymatic degradation in that it requires fewer washing steps and less hands-on time and has a fraction of the reagent costs compared to commercial alternatives (PMA ~ 0.15$/sample; QIAmp ~ 10$/sample; MolYsis ~ 7$sample; NEB ~ 30$/sample).

Any method to enrich microbial sequences will invariably have some effect on the microbial community. However, the Bray-Curtis distance between lyPMA and matched raw samples was significantly smaller than for every other host depletion method. This suggests lyPMA treatment can be used to reduce the percentage of reads in saliva samples while minimizing biases in representation in the microbial community. Importantly, the distortions induced were less than the differences among individuals, strongly suggesting that the ability to read out clinically significant microbiome states would be preserved. Furthermore, the differences observed may actually be biologically relevant. PMA treatment only detects intact, or live, microbial cells, which can have a statistically significant impact on biological interpretation, as has been shown in recent studies on a broad range of topics including the soil microbiome [23], spaceship clean rooms [24], cystic fibrosis patient samples [25], food safety [26, 27], and saliva [28].

The lyPMA method has been optimized for saliva; however, saliva is only one of many sample types where microbial analysis is hampered by a large amount of host DNA. Expanding this technique to different sample types will require tailoring the method to account for selective lysis, ideal PMA concentration, and optimal temperature and duration of light exposure.

## Conclusion

Osmotic lysis in distilled water followed by treatment with PMA (lyPMA) is a novel method to significantly reduce the percentage of human DNA in shotgun metagenomic sequencing. The method requires only standard laboratory equipment and is suitable for any DNA extraction technique. lyPMA increases microbial reads in human saliva samples by an order of magnitude. Given a low consumable cost of around 15 cents per sample, lyPMA can therefore reduce the sequencing cost by an order of magnitude.

## Methods

### qPCR evaluation of human and microbial gDNA

A total of 1 ng purified DNA from human saliva was used as a template to amplify the human-specific primer PTGER2: hPTGER2f (5′- GCTGCTTCTCATTGTCTCGG -3′) and PTGER2r (5′- GCCAGGAGAATGAGGTGGTC -3′) [19], and the 16S rRNA gene: Bakt-805R (5′- CCTACGGGNGGCWGCAG -3′) and Bakt_341f (5′- GACTACHVGGGTATCTAATCC -3′) [7]. All reactions were performed in triplicate. The final qPCR reaction volume totaled 10 μl containing 5 μl KAPA HiFi HotStart ReadyMix (2×), 1 ng DNA, 0.5 μM forward and reverse primer, 1× SYBR green (Life Technologies), and the remainder water. The qPCR amplification was carried out over 35 cycles (20 s at 98 °C, 15 s at 60 °C, 35 s at 72 °C) with an initial 3-min hot start at 95 °C and a final extension step (1 min at 72 °C). In each experiment, a standard curve was included comprising known ratios (100:0, 25:75, 50:50, 75:25, and 0:100) of human gDNA (extracted HEK293T cells) and bacterial gDNA (extracted from *Escherichia coli*) in order to extrapolate the percentage of human versus microbial DNA. All samples were run in triplicate reactions and the error bars represent standard deviation among these technical replicates.

### Preliminary attempts at host DNA removal

#### Flow cytometry

Approximately 20 mg of frozen fecal sample was homogenized with 1 ml sterile phosphate buffered saline (PBS) by vortexing at maximum speed for 10 min. The sample was centrifuged for 3 min at 2000*g*, diluted with an additional 2 ml PBS, and filtered across a 35-μm filter. Triplicate 50-μl aliquots were stored for analysis of the unsorted sample, and the remaining sample was stained with a final concentration of 2× SYBR green I in the dark for 15 min. The sample was diluted 1:10 in sterile PBS and run on a Sony SH800 FACS using a 100-μm nozzle with threshold set on the forward scatter detector at 1%. Events with SYBR-specific fluorescence emission (520 nm) stronger than vehicle control were selected for analysis. Of these SYBR-positive events, three distinct populations were gated in the forward and backward scatter axes (representing event size and density, respectively). 100,000 events per gate were sorted and centrifuged at 10,000*g* for 8 min to pellet cells. DNA was extracted from the cell pellets as detailed below.

#### Sonication

Two hundred-microliter saliva samples were sonicated in an ice bath for 15 min at 40 Hz (Branson 2510, Marshall Scientific), which was previously shown to separate microbial biofilms without lysing bacteria cells [29] and then treated with PMA as detailed below.

#### DNAse treatment

Raw saliva samples were centrifuged for 5 min at 5500*g*, and the pellet was resuspended in 100 μl 1× TURBO™ DNase buffer. Next, 3 units of TURBO™ DNAse I was added and the samples were incubated at 37 °C for 20 min. The sample was washed with 500 μl sterile 1× PBS containing 0.1 μM EDTA to inhibit the DNAse, and DNA was extracted from the pellet as detailed below.

### Comparative study details

#### Sample collection and host depletion

Volunteers were asked to refrain from eating or drinking for 1 h prior to sample collection. A total of 4 ml of unstimulated saliva was collected from eight volunteers into sterile 15-ml conical tubes. The sample was vortexed for 30 s, and 200-μl aliquots were made for each method in triplicate (18 replicate samples in total per individual). The samples were immediately processed in parallel as described below.

##### Raw (untreated)

Samples were kept on ice while the other samples were processed, then stored at − 20 °C.

##### Five-micrometer filtration (Fil)

Two hundred microliters of sterile, 1× PBS, was added to each sample and vortexed for 15 s. The diluted sample was run across a pluriStrainer® 5-μm filter (PluriSelect) by inducing low pressure with a 10-ml syringe on the Connector Ring. The effluent was retained and frozen at − 20 °C.

##### MolYsis™ Basic kit (Mol)

Samples were processed according to the manufacturer’s instructions. After removal of MolDNase A, samples were frozen at − 20 °C.

##### Qiagen QIAamp DNA Microbiome Enrichment Kit (QIA)

Samples were processed according to the manufacturer’s instructions. After proteinase K treatment, samples were frozen at − 20 °C.

##### PMA treatment (lyPMA)

Two hundred-microliter unstimulated saliva aliquots were centrifuged at 10,000*g* for 8 min. The supernatant was discarded and the cell pellet was resuspended in 200 μl sterile H_2_O by pipetting and a brief vortex then left at room temperature for 5 min to osmotically lyse mammalian cells. A final concentration of 10 μm PMA (Biotium) was added (10 μl of 0.2 mM PMA solution added to 200-μl sample), and the sample was briefly vortexed, then incubated in the dark at room temperature for 5 min. Samples were then laid horizontally on ice < 20 cm [23] from a standard, bench top fluorescent light bulb (Philips F28T5/835 ALTO 40PK) for 25 min, with brief centrifugation and rotation every ~ 5 min. After exposure, samples were frozen at − 20 °C.

##### NEBNext Microbiome DNA Enrichment Kit (NEB)

Samples were treated as raw throughout sample collection and DNA extraction, then processed according to the manufacturer’s instructions.

#### DNA extraction

Frozen samples were thawed and transferred into 96-well plates containing garnet beads and extracted using Qiagen PowerSoil DNA kit adapted for magnetic bead purification as previously described [22]. DNA was eluted in 100 μl Qiagen elution buffer.

#### Library generation and sequencing

All data presented combines two independent experiments performed identically, with each experiment containing replicate saliva samples processed as described above for four individuals each. Extracted DNA was quantified via Qubit™ dsDNA HS Assay (ThermoFisher Scientific), and 1 ng of input DNA was used in a 1:10 miniaturized Kapa HyperPlus protocol. For samples with less than 1 ng DNA, a maximum volume of 3.5 μl input was used. Library concentration was determined with triplicate readings of the Kapa Illumina Library Quantification Kit; 20 fmol of raw sample libraries and 10 fmol of host-depleted libraries were pooled and size selected for fragments between 300 and 800 bp on the Sage Science PippinHT to exclude primer dimers. The pooled library was sequenced as a paired-end 150-cycle run on an Illumina HiSeq2500 v2 in Rapid Run mode at the UCSD IGM Genomics Center.

#### Sequencing data analysis

Demultiplexed sequences were processed using an in-house modular workflow employing Snakemake [30] (https://github.com/tanaes/snakemake_assemble, commit 1c393f4). First, reads were trimmed and quality filtered using Atropos v 1.1.5, a fork of Cutadapt [31]. Reads aligning to the host genome (GRCh38.p7) were identified using Bowtie 2 v2.3.0 [32] with parameters set by the flag—very sensitive local. A total of seven samples with fewer than 50,000 quality-filtered non-human reads were excluded from downstream analysis. The host-filtered microbial reads from the remaining 137 samples were profiled using MetaPhlAn v2.0 [33] with standard parameters. The MetaPhlAn taxonomic output matrix was filtered to represent only the relative abundance of the most specific taxonomic level. Taxa only identified in one out of the 137 samples were excluded from analysis, resulting in 175 taxa. This filtered matrix was used for Bray-Curtis and Binary Jaccard beta diversity analysis using QIIME. For phylogenetic analyses including UniFrac [34], a tree was created using the MetaPhlAn2 taxonomy, with internal branches assigned a length of 1. Because some taxa could not be assigned to the tips of the tree, internal nodes were added as tips assigned a length of 0, allowing these taxa to contribute to the analysis.

## Additional files

Additional file 1: **Figure S1.** Physical approaches to separate human from microbial cells does not reduce percentage human DNA. Unless otherwise stated, evaluation of size-driven host DNA depletion methods was performed by qPCR analysis of the human-specific PTGER2 gene normalized to raw sample. A) Raw saliva was passed across a 5-μm filter, and the original sample (raw), residue left on top of the filter (res), and filtrate (fil) were compared. B) The pellet of a raw saliva sample after a 30-s centrifugation at 2500*g* (P), its supernatant (SS), the SS after pelleting all cells at 10,000*g* for 8 min (FS), and the FS pellet washed with 1× PBS (FSW) were compared. C) Distinct populations of small, medium (med), and large events by flow cytometry of a human fecal sample. D) Percentage of human DNA by shallow shotgun sequencing normalized to raw sample of distinct FACS populations from C. E) The SS of a raw saliva sample after treatment with DNAse. Significance test ordinary one-way ANOVA with Dunnett’s multiple comparisons test *p* < 0.01. (PNG 521 kb)

Additional file 2: **Figure S2.** Optimization of lyPMA conditions for human DNA depletion. qPCR analysis of the relative abundance of the human-specific PTGER2 gene normalized to raw saliva across methods of selective mammalian cell lysis (A) and PMA concentration (B). qPCR analysis of the fold change of the bacteria-specific 16S rRNA gene normalized to raw saliva across methods of selective mammalian cell lysis (C) and PMA concentration (D). SS = slow centrifugation (30 s at 2500 g), son = sonication (15 min at 60 Hz), H_2_O = osmotic lysis with pure water. (PNG 274 kb)

Additional file 3: **Figure S3.** Quality control information. A) DNA quantification pre-library-prep, but post-host-DNA-depletion. The red line indicates the concentration necessary to obtain 1 ng DNA input for library preparation given the volume limitations. B) Total number of quality filtered reads by processing method. Libraries were normalized to obtain twice as many reads for the raw samples compared to host depleted samples. C) Total number non-human reads after filtering using Bowtie 2. (PNG 299 kb)

Additional file 4: **Figure S4.** Relative abundance of the top 15 most abundant genera assigned by MetaPhlAn2 across individual and host depletion method. (PNG 803 kb)

Additional file 5: **Figure S5.** Relative taxon abundance correlation between raw and host-depleted samples. Each plot represents data from a single participant. The *x*-axis represents relative abundance in the raw sample and the *y*-axis represents relative abundance in the corresponding host depleted sample where each dot represents a distinct taxon. Error bars represent SEM across triplicate samples. The correlation values averaged across individuals for each method were not statistically different from each other (average Spearman’s rank correlation coefficient ± standard deviation: Fil = 0.789 ± 0.09, NEB = 0.75 ± 0.13, Mol = 0.82 ± 0.08, QIA = 0.83 ± 0.05, PMA = 0.82 ± 0.08) (PNG 413 kb)

Additional file 6: **Figure S6.** Host depletion via PMA treatment is possible for cryopreserved samples. Raw saliva samples were aliquoted and either frozen immediately at − 20 °C or mixed with a final concentration of 20% glycerol for cryopreservation. The percentage of human reads was assessed by Bowtie2, and the top 15 most abundant genera were assessed by MetaPhlAn2. (PNG 550 kb)
