# Supplementary figures and images for: Improving saliva shotgun metagenomics by chemical host DNA depletion
Source: Microbiome. 2018 Feb 27;6:42. doi: 10.1186/s40168-018-0426-3 (PMC5827986; doi:10.1186/s40168-018-0426-3)

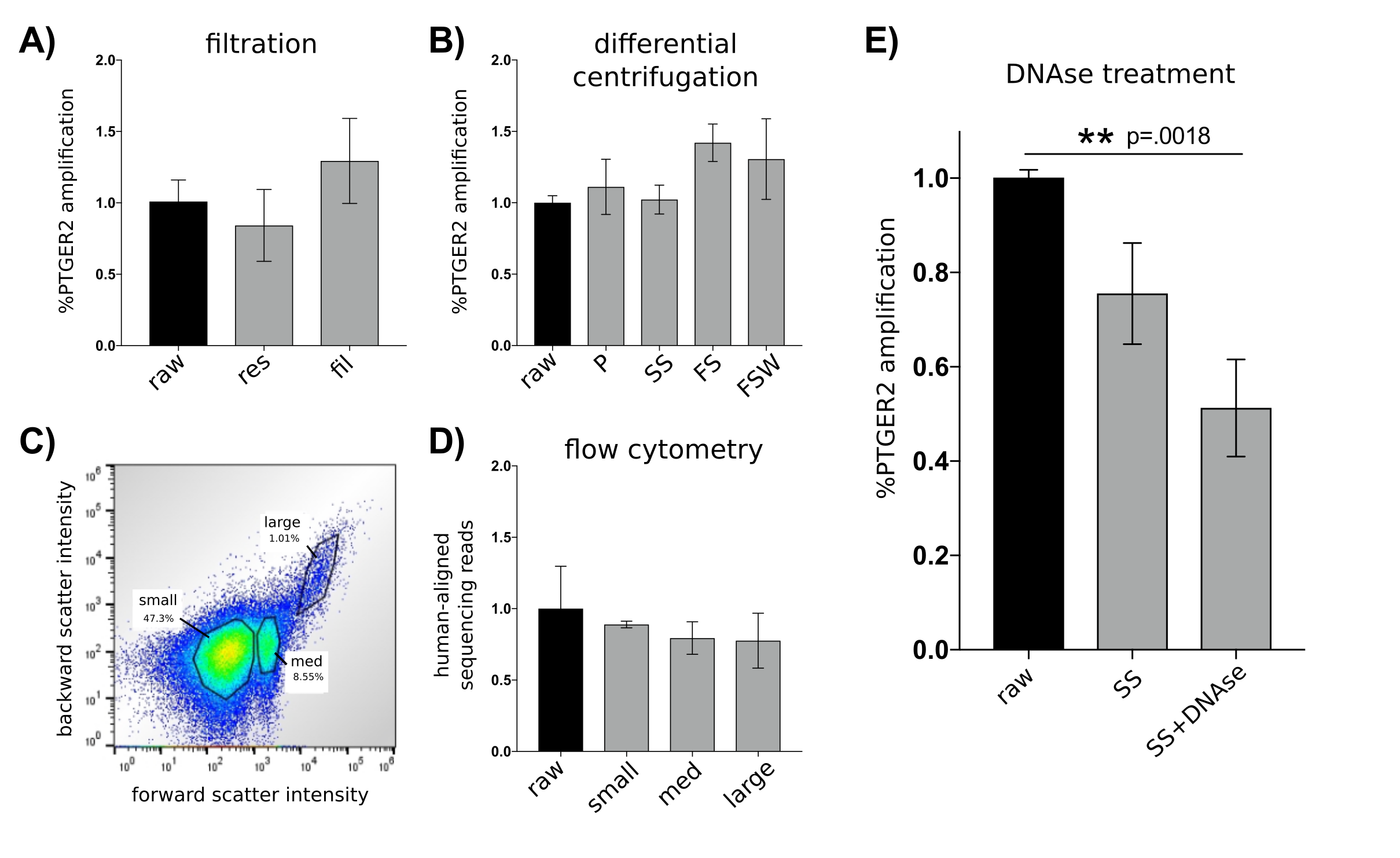

Supplement: Supplementary file 1 — Figure S1. Physical approaches to separate human from microbial cells does not reduce percentage human DNA. Unless otherwise stated, evaluation of size-driven host DNA depletion methods was performed by qPCR analysis of the human-specific PTGER2 gene normalized to raw sample. A) Raw saliva was passed across a 5-μm filter, and the original sample (raw), residue left on top of the filter (res), and filtrate (fil) were compared. B) The pellet of a raw saliva sample after a 30-s centrifugation at 2500g (P), its supernatant (SS), the SS after pelleting all cells at 10,000g for 8 min (FS), and the FS pellet washed with 1× PBS (FSW) were compared. C) Distinct populations of small, medium (med), and large events by flow cytometry of a human fecal sample. D) Percentage of human DNA by shallow shotgun sequencing normalized to raw sample of distinct FACS populations from C. E) The SS of a raw saliva sample after treatment with DNAse. Significance test ordinary one-way ANOVA with Dunnett’s multiple comparisons test p < 0.01. (PNG 521 kb) [file 40168_2018_426_MOESM1_ESM.png]

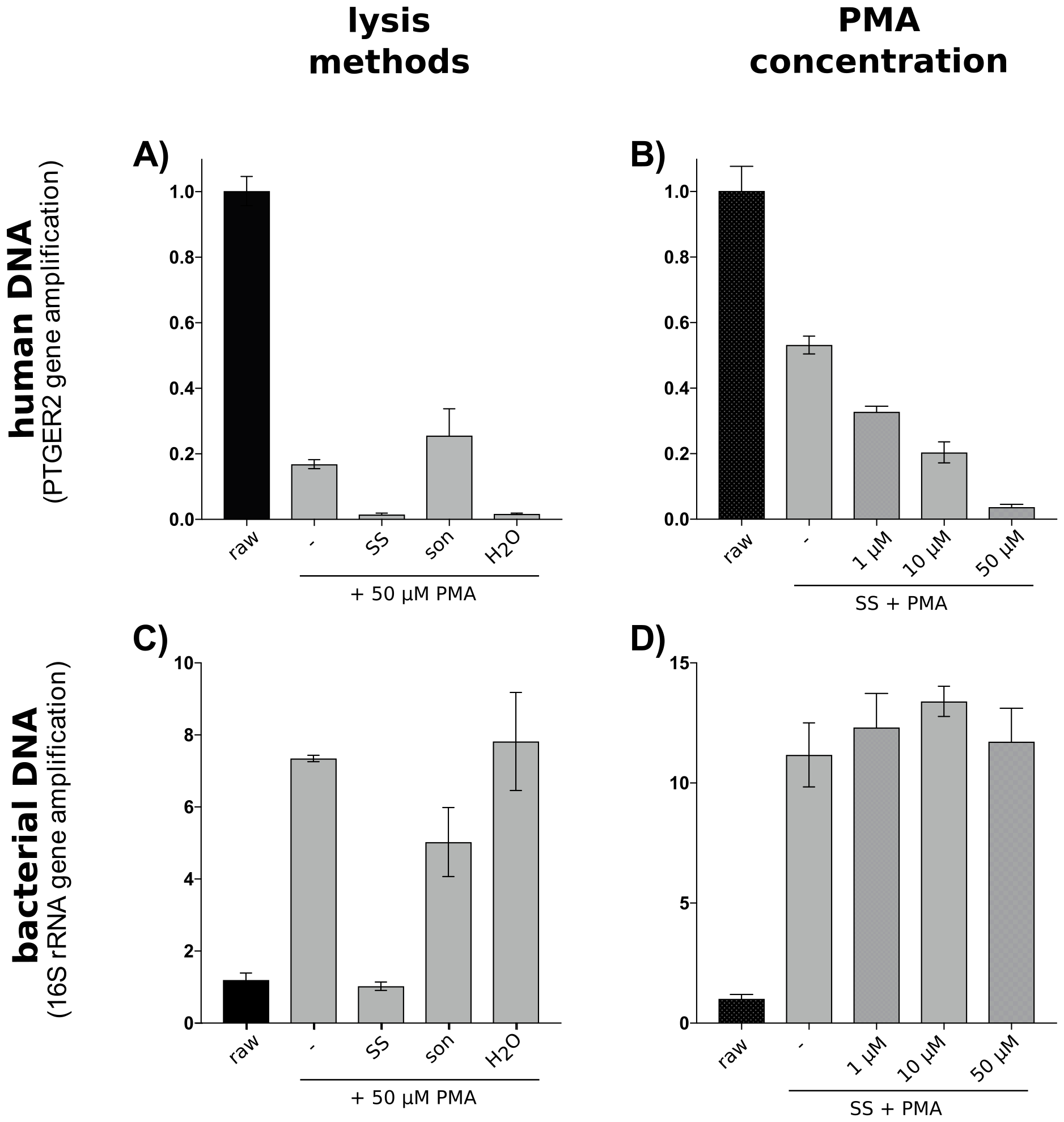

Supplement: Supplementary file 2 — Figure S2. Optimization of lyPMA conditions for human DNA depletion. qPCR analysis of the relative abundance of the human-specific PTGER2 gene normalized to raw saliva across methods of selective mammalian cell lysis (A) and PMA concentration (B). qPCR analysis of the fold change of the bacteria-specific 16S rRNA gene normalized to raw saliva across methods of selective mammalian cell lysis (C) and PMA concentration (D). SS = slow centrifugation (30 s at 2500 g), son = sonication (15 min at 60 Hz), H2O = osmotic lysis with pure water. (PNG 274 kb) [file 40168_2018_426_MOESM2_ESM.png]

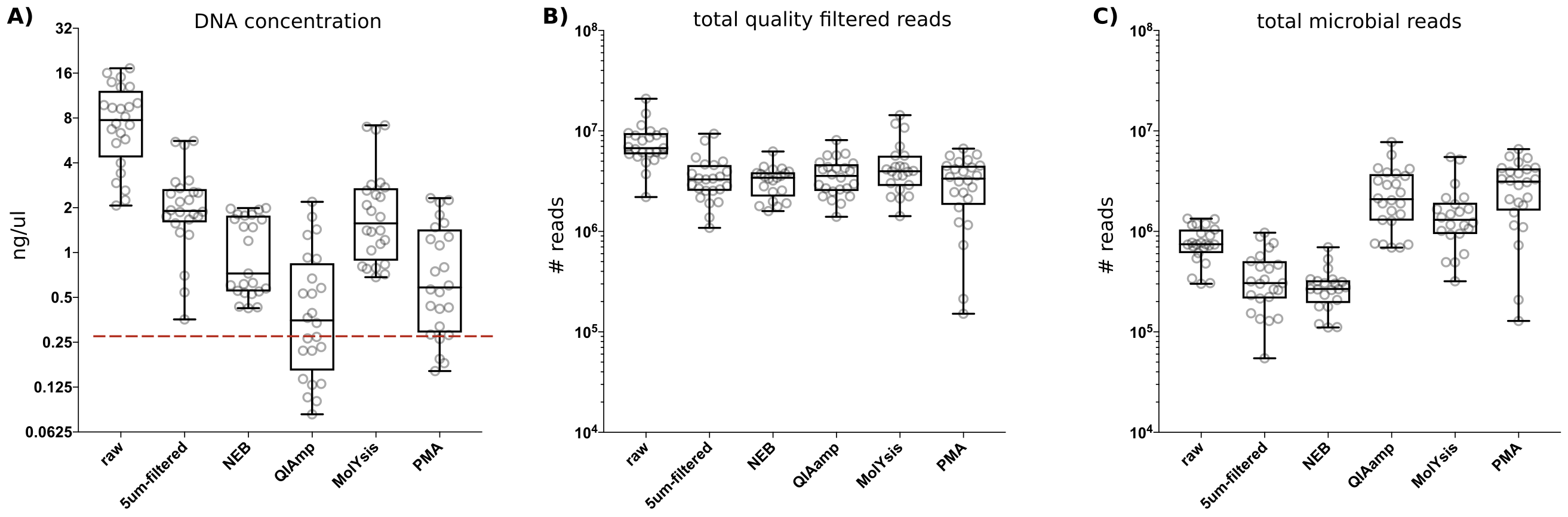

Supplement: Supplementary file 3 — Figure S3. Quality control information. A) DNA quantification pre-library-prep, but post-host-DNA-depletion. The red line indicates the concentration necessary to obtain 1 ng DNA input for library preparation given the volume limitations. B) Total number of quality filtered reads by processing method. Libraries were normalized to obtain twice as many reads for the raw samples compared to host depleted samples. C) Total number non-human reads after filtering using Bowtie 2. (PNG 299 kb) [file 40168_2018_426_MOESM3_ESM.png]

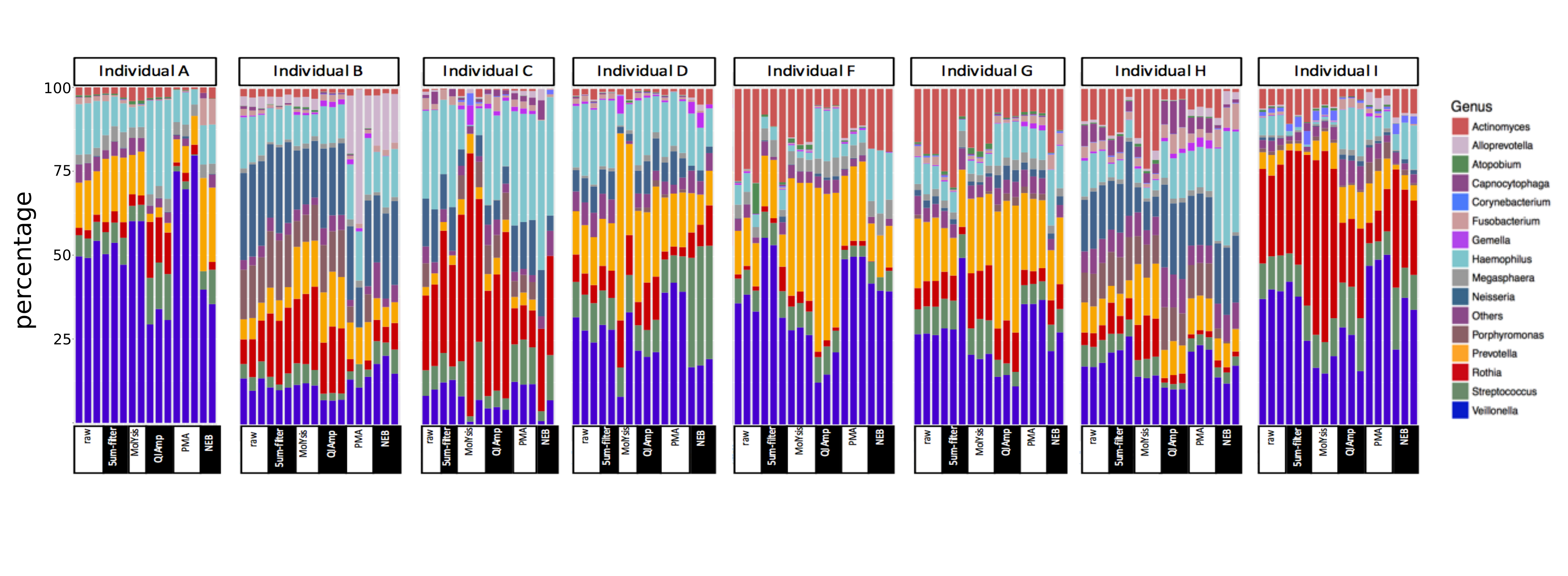

Supplement: Supplementary file 4 — Figure S4. Relative abundance of the top 15 most abundant genera assigned by MetaPhlAn2 across individual and host depletion method. (PNG 803 kb) [file 40168_2018_426_MOESM4_ESM.png]

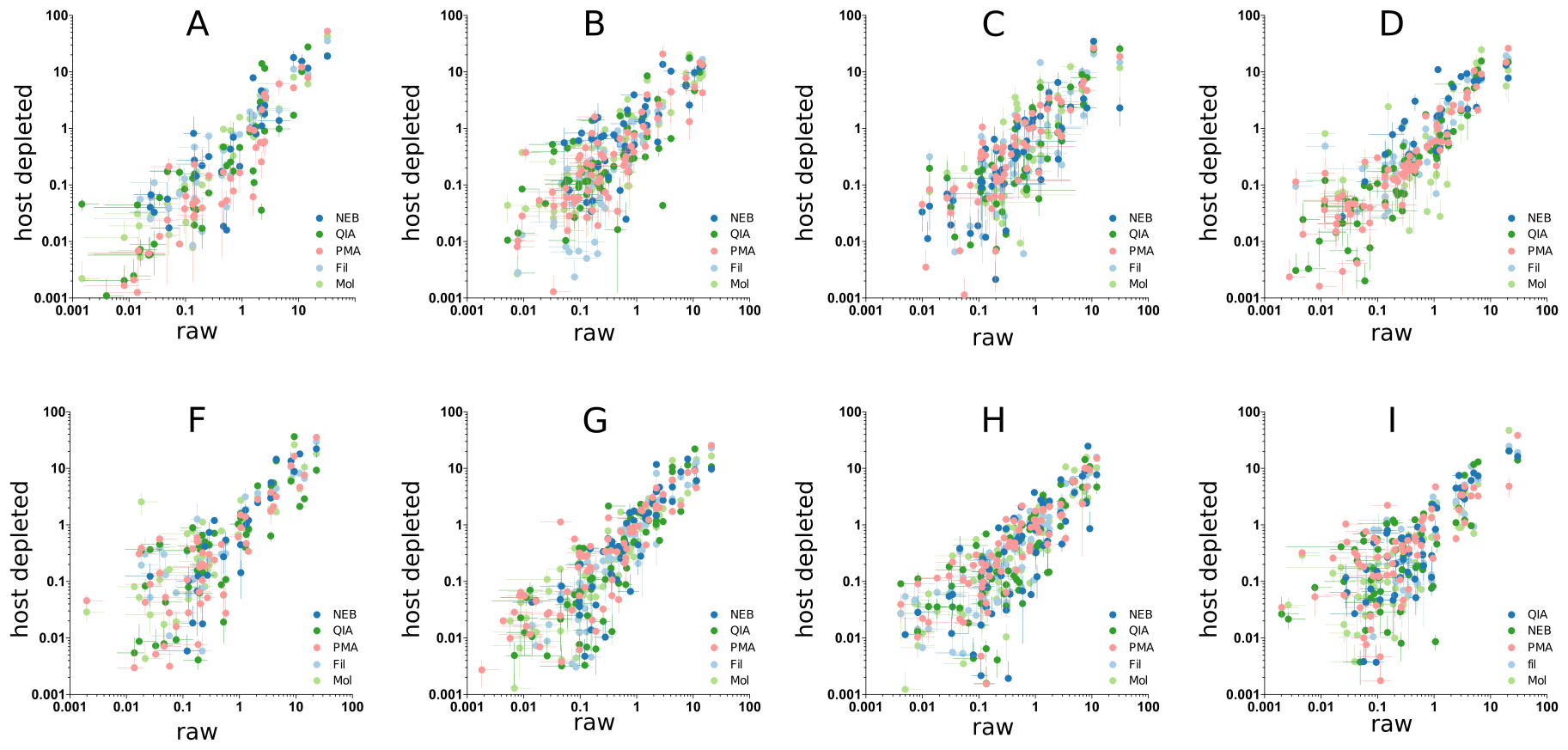

Supplement: Supplementary file 5 — Figure S5. Relative taxon abundance correlation between raw and host-depleted samples. Each plot represents data from a single participant. The x-axis represents relative abundance in the raw sample and the y-axis represents relative abundance in the corresponding host depleted sample where each dot represents a distinct taxon. Error bars represent SEM across triplicate samples. The correlation values averaged across individuals for each method were not statistically different from each other (average Spearman’s rank correlation coefficient ± standard deviation: Fil = 0.789 ± 0.09, NEB = 0.75 ± 0.13, Mol = 0.82 ± 0.08, QIA = 0.83 ± 0.05, PMA = 0.82 ± 0.08) (PNG 413 kb) [file 40168_2018_426_MOESM5_ESM.png]

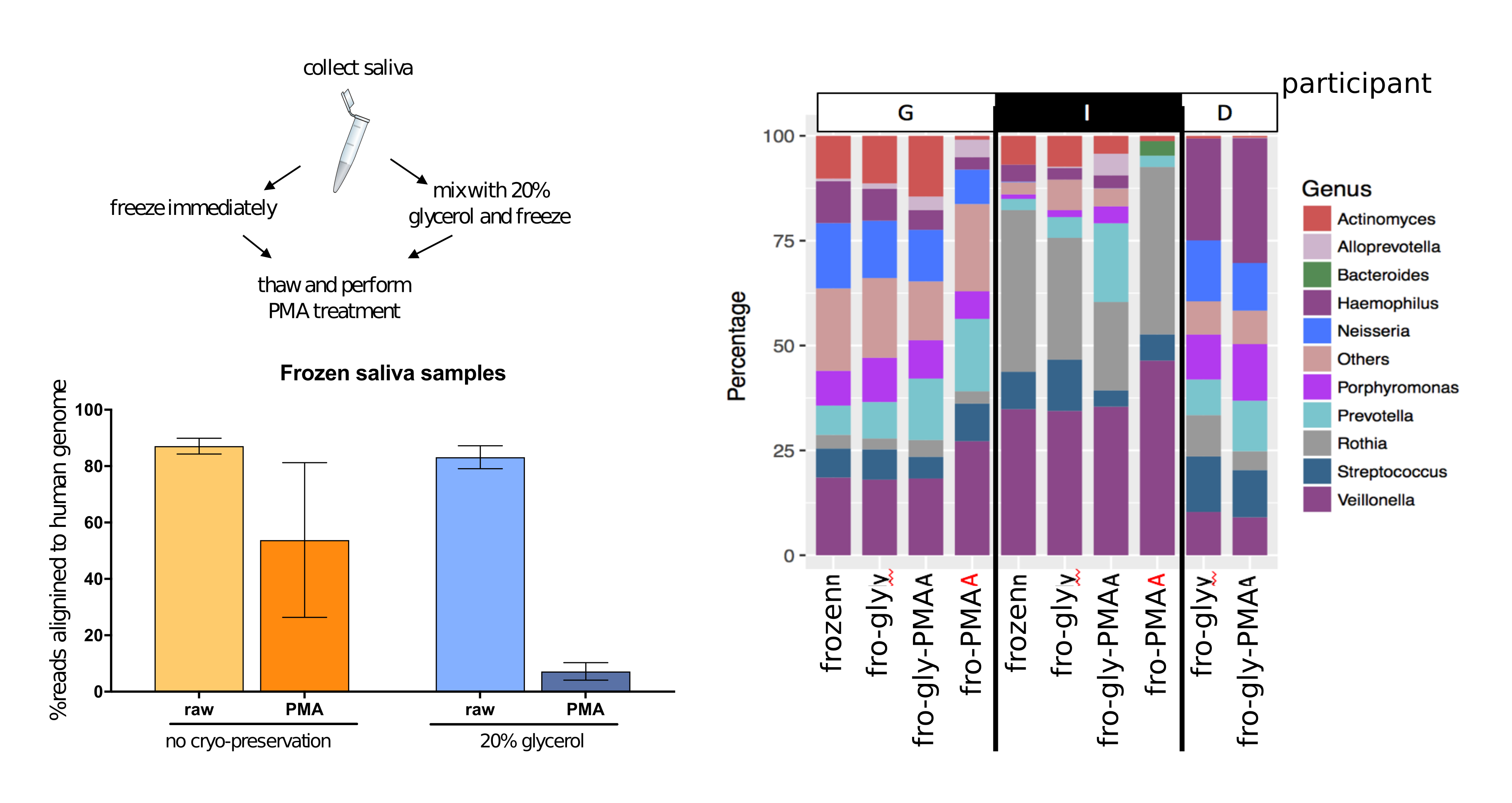

Supplement: Supplementary file 6 — Figure S6. Host depletion via PMA treatment is possible for cryopreserved samples. Raw saliva samples were aliquoted and either frozen immediately at − 20 °C or mixed with a final concentration of 20% glycerol for cryopreservation. The percentage of human reads was assessed by Bowtie2, and the top 15 most abundant genera were assessed by MetaPhlAn2. (PNG 550 kb) [file 40168_2018_426_MOESM6_ESM.png]
